# Correction: Gene regulatory patterning codes in early cell fate specification of the *C. elegans* embryo

**DOI:** 10.7554/eLife.106163

**Published:** 2025-01-27

**Authors:** Alison G Cole, Tamar Hashinshony, Zhuo Du, Itai Yanai

**Keywords:** *C. elegans*

Cole AG, Hashimshony T, Du Z, Yanai I. 2024. Gene regulatory patterning codes in early cell fate specification of the *C. elegans* embryo. *eLife* **12**:RP87099. doi: 10.7554/eLife.87099.

Published 29 January 2024

Due to a typological error in the analysis code, cell ID ABpxpap was mis-labelled in the Supplementary file 5 and 6 as ABxpap. This typographical error does not appear in the main text. We are grateful to Ahmed Elewa who noticed the error and reported it to us. We apologise for any inconvenience.

Incorrect text:

ABxpap

Corrected text:

ABpxpap

The article has been corrected accordingly.

