# Supplementary material for: Gene regulatory patterning codes in early cell fate specification of the C. elegans embryo
Source: eLife. 2024 Jan 29;12:RP87099. doi: 10.7554/eLife.87099 (PMC10945703; doi:10.7554/eLife.87099)
Supplement: Supplementary file 1. — Rows correspond to a studied embryo. The average number of transcripts and genes detected are shown. [file elife-87099-supp1.docx]

**Table S1:** Characteristics of the dataset. Rows correspond to a studied embryo. The average number of transcripts and genes detected are shown.

| Embryo ID | # Cells Collected | Intended Stage | # Transcripts | # Genes |
| --- | --- | --- | --- | --- |
| 1.1 | 1 | 1 | 501109 | 7110 |
| 1.2 | 1 | 1 | 145605 | 6138 |
| 1.3 | 1 | 1 | 245833 | 6605 |
| 2.1 | 2 | 2 | 90003 | 6337 |
| 2.3 | 2 | 2 | 79524 | 6249 |
| 2.4 | 2 | 2 | 69301 | 6110 |
| 2.5 | 2 | 2 | 76010 | 6186 |
| 4.1 | 4 | 4 | 57704 | 6827 |
| 4.3 | 4 | 4 | 52980 | 6603 |
| 4.5 | 4 | 4 | 502699 | 7776 |
| 7.1 | 7 | 8 | 256118 | 9980 |
| 8.1 | 7 | 8 | 270101 | 10102 |
| 8.2 | 6 | 8 | 251105 | 9525 |
| 8.3 | 7 | 8 | 243433 | 9938 |
| 8.4 | 8 | 8 | 275563 | 9819 |
| 10.1 | 10 | 8 | 244450 | 10501 |
| 13 | 11 | 15 | 121911 | 10600 |
| 13.2 | 11 | 15 | 146248 | 10173 |
| 14.2 | 14 | 15 | 183105 | 11323 |
| 15 | 11 | 15 | 143850 | 10532 |
| 17.2 | 17 | 15 | 138718 | 11363 |
| 22 | 21 | 28 | 149171 | 12485 |
| 23 | 20 | 28 | 129557 | 12078 |
| 24.1 | 24 | 28 | 139130 | 11762 |
| 24.2 | 23 | 28 | 91587 | 11562 |
| 25 | 23 | 28 | 82082 | 11406 |
| 27 | 26 | 28 | 113177 | 12007 |
| 45 | 41 | 51 | 60949 | 11840 |
| 48 | 48 | 51 | 51712 | 12066 |
| 48.2 | 42 | 51 | 35261 | 10977 |
| 51 | 38 | 51 | 42480 | 11675 |
| 55 | 53 | 51 | 48942 | 12409 |
| 72 | 68 | 102 | 44563 | 12249 |
| 82 | 76 | 102 | 38586 | 11743 |
| 88 | 83 | 102 | 38371 | 11874 |
| 92 | 90 | 102 | 39739 | 11900 |
| 96 | 96 | 102 | 44019 | 11855 |
| 96.2 | 75 | 102 | 26029 | 11555 |
