# Supplementary material for: Gene regulatory patterning codes in early cell fate specification of the C. elegans embryo
Source: eLife. 2024 Jan 29;12:RP87099. doi: 10.7554/eLife.87099 (PMC10945703; doi:10.7554/eLife.87099)
Supplement: Supplementary file 2. — Cell state markers for the expression profiles used for assignment were curated from the literature (da Veiga Beltrame et al., 2022) and, in particular, one large study (Murray et al., 2012). [file elife-87099-supp2.docx]

**Table S2: Gene markers from the literature.** Cell state markers for the expression profiles used for assignment were curated from the literature [(da Veiga Beltrame et al., 2022)](https://paperpile.com/c/M2W8QA/LDCJ) and, in particular, one large study [(Murray et al., 2012)](https://paperpile.com/c/M2W8QA/AyCt).

| Gene | PMID | Title | Reference |
| --- | --- | --- | --- |
| *mex-3* | 8861905 | MEX-3 is a KH domain protein that regulates blastomere identity in early C. elegans embryos. | Cell. 1996 Oct 18;87(2):205-16. |
| *oma-1* | 16611242 | OMA-1 is a P granules-associated protein that is required for germline specification in Caenorhabditis elegans embryos. | Genes Cells. 2006 Apr;11(4):383-96 |
| *pes-10* | 7607073 | Soma-germline asymmetry in the distributions of embryonic RNAs in Caenorhabditis elegans. | Development. 1994 Oct;120(10):2823-34. |
| *cey-3* | 25487147 | Spatiotemporal transcriptomics reveals the evolutionary history of the endoderm germ layer. | Nature. 2015 Mar 12;519(7542):219-22 |
| *end-3* | 12142026 | Dynamics of a developmental switch: recursive intracellular and intranuclear redistribution of Caenorhabditis elegans POP-1 parallels Wnt-inhibited transcriptional repression. | Dev Biol. 2002 Aug 1;248(1):128-42. |
| *hlh-26* | 15935776 | The REF-1 family of bHLH transcription factors pattern C. elegans embryos through Notch-dependent and Notch-independent pathways. | Dev Cell. 2005 Jun;8(6):867-79. |
| *mom-2* | 9288749 | Wnt signaling polarizes an early C. elegans blastomere to distinguish endoderm from mesoderm. | Cell. 1997 Aug 22;90(4):695-705. |
| *tbx-35* | 16831832 | Specification of the C. elegans MS blastomere by the T-box factor TBX-35 | Development. 2006 Aug;133(16):3097-106. |
| *tbx-37* | 15056620 | The T-box transcription factors TBX-37 and TBX-38 link GLP-1/Notch signaling to mesoderm induction in C. elegans embryos. | Development. 2004 May;131(9):1967-78. |
| *tbx-38* | 15056620 | The T-box transcription factors TBX-37 and TBX-38 link GLP-1/Notch signaling to mesoderm induction in C. elegans embryos. | Development. 2004 May;131(9):1967-78. |
| *ceh-13* | 9334268 | The expression of the C. elegans labial-like Hox gene ceh-13 during early embryogenesis relies on cell fate and on anteroposterior cell polarity. | Development. 1997 Nov;124(21):4193-200. |
| *ceh-51* | 19605496 | The NK-2 class homeodomain factor CEH-51 and the T-box factor TBX-35 have overlapping function in C. elegans mesoderm development. | Development. 2009 Aug;136(16):2735-46. |
| *cey-3* | 25487147 | Spatiotemporal transcriptomics reveals the evolutionary history of the endoderm germ layer. | Nature. 2015 Mar 12;519(7542):219-22 |
| *end-1* | 12142026 | Dynamics of a developmental switch: recursive intracellular and intranuclear redistribution of Caenorhabditis elegans POP-1 parallels Wnt-inhibited transcriptional repression. | Dev Biol. 2002 Aug 1;248(1):128-42. |
| *end-3* | 12142026 | Dynamics of a developmental switch: recursive intracellular and intranuclear redistribution of Caenorhabditis elegans POP-1 parallels Wnt-inhibited transcriptional repression. | Dev Biol. 2002 Aug 1;248(1):128-42. |
| *F19F10.1* | Data | Figure 2 | This work |
| *hlh-26* | 15935776 | The REF-1 family of bHLH transcription factors pattern C. elegans embryos through Notch-dependent and Notch-independent pathways. | Dev Cell. 2005 Jun;8(6):867-79. |
| *pal-1* | 11133155 | Zygotic expression of the caudal homolog pal-1 is required for posterior patterning in Caenorhabditis elegans embryogenesis. | Dev Biol. 2001 Jan 1;229(1):71-88. |
| *ref-1* | 15935776 | The REF-1 family of bHLH transcription factors pattern C. elegans embryos through Notch-dependent and Notch-independent pathways. | Dev Cell. 2005 Jun;8(6):867-79. |
| *tbx-38* | 15056620 | The T-box transcription factors TBX-37 and TBX-38 link GLP-1/Notch signaling to mesoderm induction in C. elegans embryos. | Development. 2004 May;131(9):1967-78. |
| *ceh-13* | 9334268 | The expression of the C. elegans labial-like Hox gene ceh-13 during early embryogenesis relies on cell fate and on anteroposterior cell polarity. | Development. 1997 Nov;124(21):4193-200. |
| *ceh-32* | 22508763 | Multidimensional regulation of gene expression in the C. elegans embryo | http://epic.gs.washington.edu/Epic2/ |
| *ceh-43* | 22508763 | Multidimensional regulation of gene expression in the C. elegans embryo | http://epic.gs.washington.edu/Epic2/ |
| *ceh-51* | 19605496 | The NK-2 class homeodomain factor CEH-51 and the T-box factor TBX-35 have overlapping function in C. elegans mesoderm development. | Development. 2009 Aug;136(16):2735-46. |
| *cey-3* | 25487147 | Spatiotemporal transcriptomics reveals the evolutionary history of the endoderm germ layer. | Nature. 2015 Mar 12;519(7542):219-22 |
| *dve-1* | 22508763 | Multidimensional regulation of gene expression in the C. elegans embryo. | Genome Res. 2012 Jul;22(7):1282-94. |
| *elt-1* | 22508763 | Multidimensional regulation of gene expression in the C. elegans embryo | http://epic.gs.washington.edu/Epic2/ |
| *ets-7* | 22508763 | Multidimensional regulation of gene expression in the C. elegans embryo | http://epic.gs.washington.edu/Epic2/ |
| *hlh-14* | 22508763 | Multidimensional regulation of gene expression in the C. elegans embryo |  |
| *hnd-1* | 22508763 | Multidimensional regulation of gene expression in the C. elegans embryo | http://epic.gs.washington.edu/Epic2/ |
| *lim-7* | 22508763 | Multidimensional regulation of gene expression in the C. elegans embryo |  |
| *ngn-1* | 22508763 | Multidimensional regulation of gene expression in the C. elegans embryo | http://epic.gs.washington.edu/Epic2/ |
| *nob-1* | 22508763 | Multidimensional regulation of gene expression in the C. elegans embryo. | Genome Res. 2012 Jul;22(7):1282-94. |
| *pal-1* | 11133155 | Zygotic expression of the caudal homolog pal-1 is required for posterior patterning in Caenorhabditis elegans embryogenesis. | Dev Biol. 2001 Jan 1;229(1):71-88. |
| *pes-1* | 22508763 | Multidimensional regulation of gene expression in the C. elegans embryo | http://epic.gs.washington.edu/Epic2/ |
| *pha-4* | 9649499 | pha-4, an HNF-3 homolog, specifies pharyngeal organ identity in Caenorhabditis elegans. | Genes Dev. 1998 Jul 1;12(13):1947-52. |
| *ref-1* | 22508763 | Multidimensional regulation of gene expression in the C. elegans embryo | http://epic.gs.washington.edu/Epic2/ |
| *tbx-38* | 15056620 | The T-box transcription factors TBX-37 and TBX-38 link GLP-1/Notch signaling to mesoderm induction in C. elegans embryos. | Development. 2004 May;131(9):1967-78. |
| *tbx-8* | 22508763 | Multidimensional regulation of gene expression in the C. elegans embryo | http://epic.gs.washington.edu/Epic2/ |
| *unc-30* | 25738873 | The Bicoid Class Homeodomain Factors ceh-36/OTX and unc-30/PITX Cooperate in C. elegans Embryonic Progenitor Cells to Regulate Robust Development. | PLoS Genet 11(3): e1005003 |
| *ceh-13* | 9334268 | The expression of the C. elegans labial-like Hox gene ceh-13 during early embryogenesis relies on cell fate and on anteroposterior cell polarity. | Development. 1997 Nov;124(21):4193-200. |
| *ceh-16* | 15659483 | Ceh-16/engrailed patterns the embryonic epidermis of Caenorhabditis elegans | Development 132(4):739-49 |
| *ceh-27* | 22508763 | Multidimensional regulation of gene expression in the C. elegans embryo | http://epic.gs.washington.edu/Epic2/ |
| *ceh-32* | 22508763 | Multidimensional regulation of gene expression in the C. elegans embryo | http://epic.gs.washington.edu/Epic2/ |
| *ceh-36* | 25738873 | The Bicoid Class Homeodomain Factors ceh-36/OTX and unc-30/PITX Cooperate in C. elegans Embryonic Progenitor Cells to Regulate Robust Development. | PLoS Genet 11(3): e1005003 |
| *ceh-43* | 22508763 | Multidimensional regulation of gene expression in the C. elegans embryo | http://epic.gs.washington.edu/Epic2/ |
| *ceh-51* | 19605496 | The NK-2 class homeodomain factor CEH-51 and the T-box factor TBX-35 have overlapping function in C. elegans mesoderm development. | Development. 2009 Aug;136(16):2735-46. |
| *elt-1* | 22508763 | Multidimensional regulation of gene expression in the C. elegans embryo | http://epic.gs.washington.edu/Epic2/ |
| *elt-7* | 22508763 | Multidimensional regulation of gene expression in the C. elegans embryo | http://epic.gs.washington.edu/Epic2/ |
| *hlh-1* | 22508763 | Multidimensional regulation of gene expression in the C. elegans embryo | http://epic.gs.washington.edu/Epic2/ |
| *hlh-2* | 22508763 | Multidimensional regulation of gene expression in the C. elegans embryo | http://epic.gs.washington.edu/Epic2/ |
| *hlh-3* | 22508763 | Multidimensional regulation of gene expression in the C. elegans embryo | http://epic.gs.washington.edu/Epic2/ |
| *irx-1* | 22508763 | Multidimensional regulation of gene expression in the C. elegans embryo | http://epic.gs.washington.edu/Epic2/ |
| *lag-2* | 22508763 | Multidimensional regulation of gene expression in the C. elegans embryo | http://epic.gs.washington.edu/Epic2/ |
| *lin-32* | 22508763 | Multidimensional regulation of gene expression in the C. elegans embryo | http://epic.gs.washington.edu/Epic2/ |
| *nob-1* | 22508763 | Multidimensional regulation of gene expression in the C. elegans embryo | http://epic.gs.washington.edu/Epic2/ |
| *nos-2* | 10518502 | nos-1 and nos-2, two genes related to Drosophila nanos, regulate primordial germ cell development and survival in Caenorhabditis elegans | DevelopmentVolume 126, Issue 21, November 1999, Pages 4861-4871 |
| *pal-1* | 11133155 | Zygotic expression of the caudal homolog pal-1 is required for posterior patterning in Caenorhabditis elegans embryogenesis. | Dev Biol. 2001 Jan 1;229(1):71-88. |
| *pax-3* | 22508763 | Multidimensional regulation of gene expression in the C. elegans embryo | http://epic.gs.washington.edu/Epic2/ |
| *pes-1* | 22508763 | Multidimensional regulation of gene expression in the C. elegans embryo | http://epic.gs.washington.edu/Epic2/ |
| *pha-4* | 22508763 | Multidimensional regulation of gene expression in the C. elegans embryo | http://epic.gs.washington.edu/Epic2/ |
| *ref-1* | 15935776 | The REF-1 family of bHLH transcription factors pattern C. elegans embryos through Notch-dependent and Notch-independent pathways. | Dev Cell. 2005 Jun;8(6):867-79. |
| *ref-2* | 22508763 | Multidimensional regulation of gene expression in the C. elegans embryo | http://epic.gs.washington.edu/Epic2/ |
| *tbx-11* | 22508763 | Multidimensional regulation of gene expression in the C. elegans embryo | http://epic.gs.washington.edu/Epic2/ |
| *tbx-8* | 22508763 | Multidimensional regulation of gene expression in the C. elegans embryo | http://epic.gs.washington.edu/Epic2/ |
| *tbx-9* | 22508763 | Multidimensional regulation of gene expression in the C. elegans embryo | http://epic.gs.washington.edu/Epic2/ |
| *unc-120* | 22508763 | Multidimensional regulation of gene expression in the C. elegans embryo | http://epic.gs.washington.edu/Epic2/ |
| *unc-130* | 22508763 | Multidimensional regulation of gene expression in the C. elegans embryo | http://epic.gs.washington.edu/Epic2/ |
| *vab-7* | 22508763 | Multidimensional regulation of gene expression in the C. elegans embryo | http://epic.gs.washington.edu/Epic2/ |
| *ztf-11* | 22508763 | Multidimensional regulation of gene expression in the C. elegans embryo | http://epic.gs.washington.edu/Epic2/ |
