# Supplementary material for: Gene regulatory patterning codes in early cell fate specification of the C. elegans embryo
Source: eLife. 2024 Jan 29;12:RP87099. doi: 10.7554/eLife.87099 (PMC10945703; doi:10.7554/eLife.87099)
Supplement: Supplementary file 4. — Each row corresponds to 1 of the 119 inferred cell states. The third column indicates the number of samples (scRNA-Seq cells) annotated to each cell state. [file elife-87099-supp4.docx]

**Table S4: Inferred cell states.** Each row corresponds to one of the 119 inferred cell states. The third column indicates the number of samples (scRNA-Seq cells) annotated to each cell state.

| Cell-state | Cell identities | # cells |
| --- | --- | --- |
| 1 | P_0 | 3 |
| 2 | AB | 4 |
| 3 | P1 | 4 |
| 4 | ABa | 3 |
| 5 | ABp | 3 |
| 6 | EMS | 3 |
| 7 | P2 | 3 |
| 8 | ABal ABar | 12 |
| 9 | ABpl ABpr | 11 |
| 10 | MS | 5 |
| 11 | E | 6 |
| 12 | C | 4 |
| 13 | P3 (8-cell stage) | 5 |
| 14 | ABala | 4 |
| 15 | ABalp | 5 |
| 16 | ABara | 4 |
| 17 | ABarp | 4 |
| 18 | ABpla ABpra | 9 |
| 19 | ABplp ABprp | 10 |
| 20 | MSa MSp | 12 |
| 21 | Ea_15 | 3 |
| 22 | Ep_15 | 4 |
| 23 | Ca Cp | 6 |
| 24 | P3 (15-cell stage) | 4 |
| 25 | ABalaa | 3 |
| 26 | ABalap | 5 |
| 27 | ABalpa | 6 |
| 28 | ABalpp | 4 |
| 29 | ABaraa | 4 |
| 30 | ABarap | 5 |
| 31 | ABarpa | 4 |
| 32 | ABarpp | 6 |
| 33 | ABplaa ABpraa | 12 |
| 34 | ABplap ABprap | 11 |
| 35 | ABplpa ABprpa | 13 |
| 36 | ABplpp ABprpp | 11 |
| 37 | MSaa MSpa | 8 |
| 38 | MSap MSpp | 5 |
| 39 | Ea (28-cell stage) | 4 |
| 40 | Ep (28-cell stage) | 5 |
| 41 | Caa Cpa | 7 |
| 42 | Cap Cpp | 8 |
| 43 | D | 5 |
| 44 | P4 (28-cell stage) | 6 |
| 45 | ABalaaa | 3 |
| 46 | ABalaap | 4 |
| 47 | ABalapa | 5 |
| 48 | ABalapp | 4 |
| 49 | ABalpaa | 5 |
| 50 | ABalpap | 4 |
| 51 | ABalppa | 4 |
| 52 | ABalppp | 3 |
| 53 | ABaraaa | 4 |
| 54 | ABaraap | 5 |
| 55 | ABarapa | 5 |
| 56 | ABarapp | 3 |
| 57 | ABarpaa ABarpap | 9 |
| 58 | ABarppa ABarppp | 7 |
| 59 | ABplaaa ABpraaa | 10 |
| 60 | ABplaap ABpraap | 10 |
| 61 | ABplapa ABprapa | 11 |
| 62 | ABplapp ABprapp | 7 |
| 63 | ABplpaa ABprpaa | 9 |
| 64 | ABplpap ABprpap | 8 |
| 65 | ABplppa ABprppa | 8 |
| 66 | ABplppp ABprppp | 6 |
| 67 | MSaaa | 6 |
| 68 | MSaap | 3 |
| 69 | MSapa | 2 |
| 70 | MSapp | 5 |
| 71 | MSpaa | 3 |
| 72 | MSpap | 2 |
| 73 | MSppa | 5 |
| 74 | MSppp | 4 |
| 75 | Eal Ear | 9 |
| 76 | Epl Epr | 5 |
| 77 | Caa (51-cell stage) | 6 |
| 78 | Cap (51-cell stage) | 3 |
| 79 | Cpa (51-cell stage) | 6 |
| 80 | Cpp (51-cell stage) | 3 |
| 81 | Da Dp | 4 |
| 82 | P4 (51-cell stage) | 4 |
| 83 | ABalaaaa ABalaaap | 14 |
| 84 | ABalaapa ABalaapp | 12 |
| 85 | ABalapaa ABalapap | 13 |
| 86 | ABalappa ABalappp | 15 |
| 87 | ABalpaaa ABalpaap | 6 |
| 88 | ABalpapa ABalpapp | 10 |
| 89 | ABalppaa ABalppap | 9 |
| 90 | ABalpppa ABalpppp | 11 |
| 91 | ABaraaaa ABaraaap | 8 |
| 92 | ABaraapa ABaraapp | 6 |
| 93 | ABarapaa ABarapap | 10 |
| 94 | ABarappa ABarappp | 4 |
| 95 | ABarpaaa ABarpaap ABarpapa ABarpapp | 17 |
| 96 | ABarppaa ABarppap | 14 |
| 97 | ABarpppa ABarpppp | 14 |
| 98 | ABplaaaa ABplaaap ABpraaaa ABpraaap | 3 |
| 99 | ABplaapa ABplaapp ABpraapa ABpraapp | 12 |
| 100 | ABplapaa ABplapap ABprapaa ABprapap | 11 |
| 101 | ABplappa ABplappp ABprappa ABprappp | 21 |
| 102 | ABplpaaa ABplpaap ABprpaaa ABprpaap | 10 |
| 103 | ABplpapa ABplpapp ABprpapa ABprpapp | 17 |
| 104 | ABplppaa ABplppap ABprppaa ABprppap | 6 |
| 105 | ABplpppa ABplpppp ABprpppa ABprpppp | 12 |
| 106 | MSaaaa MSpaaa | 9 |
| 107 | MSaaap MSaapp MSpaap MSpapp | 9 |
| 108 | MSaapa MSpapa | 8 |
| 109 | MSapaa MSapap MSppaa MSppap | 10 |
| 110 | MSappa MSpppa | 7 |
| 111 | MSappp MSpppp | 11 |
| 112 | Eala Ealp Eara Earp | 19 |
| 113 | Epla Eplp Epra Eprp | 9 |
| 114 | Caaa Caap | 5 |
| 115 | Capa Capp | 7 |
| 116 | Cpaa Cpap | 4 |
| 117 | Cppa Cppp | 4 |
| 118 | Daa Dap Dpa Dpp | 14 |
| 119 | P4a P4p (102-cell stage) | 8 |
